# The DIAD Approach to Correlative Synchrotron X-ray Imaging and Diffraction Analysis of Human Enamel

**DOI:** 10.1021/cbmi.3c00122

**Published:** 2024-03-08

**Authors:** Cyril Besnard, Ali Marie, Sisini Sasidharan, Hans Deyhle, Andrew M. James, Sharif I. Ahmed, Christina Reinhard, Robert A. Harper, Richard M. Shelton, Gabriel Landini, Alexander M. Korsunsky

**Affiliations:** † Department of Engineering Science, University of Oxford, Oxford, Oxfordshire OX1 3PJ, United Kingdom; ‡ Diamond Light Source Ltd., Didcot, Oxfordshire OX11 0DE, United Kingdom; § School of Dentistry, University of Birmingham, 5 Mill Pool Way, Edgbaston, Birmingham, West Midlands B5 7EG, United Kingdom; ∥ Trinity College, University of Oxford, Broad Street, Oxford, Oxfordshire OX1 3BH, United Kingdom

**Keywords:** human enamel, dental caries, correlative analysis, synchrotron, tomography, diffraction

## Abstract

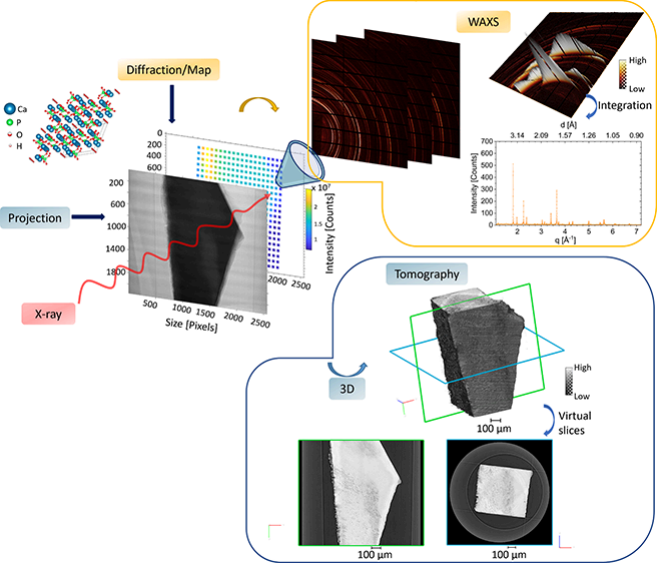

The Dual Imaging and Diffraction (DIAD) beamline at Diamond Light Source (Didcot, U.K.) implements a correlative approach to the dynamic study of materials based on concurrent analysis of identical sample locations using complementary X-ray modalities to reveal structural detail at various length scales. Namely, the underlying beamline principle and its practical implementation allow the collocation of chosen regions within the sample and their interrogation using real-space imaging (radiography and tomography) and reciprocal space scattering (diffraction). The switching between the two principal modes is made smooth and rapid by design, so that the data collected is interlaced to obtain near-simultaneous multimodal characterization. Different specific photon energies are used for each mode, and the interlacing of acquisition steps allows conducting static and dynamic experiments. Building on the demonstrated realization of this state-of-the-art approach requires further refining of the experimental practice, namely, the methods for gauge volume collocation under different modes of beam–sample interaction. To address this challenge, experiments were conducted at DIAD devoted to the study of human dental enamel, a hierarchical structure composed of hydroxyapatite mineral nanocrystals, as a static sample previously affected by dental caries (tooth decay) as well as under dynamic conditions simulating the process of acid demineralization. Collocation and correlation were achieved between WAXS (wide-angle X-ray scattering), 2D (radiographic), and 3D (tomographic) imaging. While X-ray imaging in 2D or 3D modes reveals real-space details of the sample microstructure, X-ray scattering data for each gauge volume provided statistical nanoscale and ultrastructural polycrystal reciprocal-space information such as phase and preferred orientation (texture). Careful registration of the gauge volume positions recorded during the scans allowed direct covisualization of the data from two modalities. Diffraction gauge volumes were identified and visualized within the tomographic data sets, revealing the underlying local information to support the interpretation of the diffraction patterns. The present implementation of the 4D microscopy paradigm allowed following the progression of demineralization and its correlation with time-dependent WAXS pattern evolution in an approach that is transferable to other material systems.

## Introduction

The correlative imaging paradigm aims to collect data using different modalities at matched locations to extract rich multimodal information.^1−5^ However, the practical application of this idea presents significant challenges.^1,6^ Synchrotron instruments are eminently well-suited for this type of study,^7−10^ but the practical realization of the correlative approach requires both hardware and software implementation and the advancement of practical experimental techniques. The principal point of interest in conducting experiments at the recently commissioned Dual Imaging and Diffraction (DIAD)^11−13^ beamline at Diamond Light Source (DLS) is to enable concurrent data collection in two modalities without changes to the beamline setup: the optics train, beam energies, and detector positions. The DIAD beamline entered early user operation in 2021 and offers a configuration that allows imaging and diffraction data collection with fast changes between acquisition modes. A key step during the experiment setup is the registration to match the measurement acquisition positions for both modes. Notably, the diffraction mode may utilize the beam energy and bandwidth combination that differs from the other techniques, such as full-field tomography, that often benefit from using a polychromatic (pink) beam with a broader energy spread.^14^

As described by Reinhard et al.,^11^ DIAD splits the primary beam into independent imaging and diffraction branches, each equipped with its own optics train. The branches then intercept one another at the sample experiment location. Fast gating of beams and dedicated detectors enable virtually simultaneous (concurrent) data collection in two modes. In the resulting setup, wide-angle X-ray scattering (WAXS) extracts reciprocal space pico- to nanoscale structural information, while the imaging and tomography branch provides real-space imaging at the nano- to microscale.^15−18^

Using human enamel as the study case, we present a method to identify and visualize the probe (gauge volume) for the diffraction mode, demonstrating the advantages of correlative analysis at DIAD. Dental enamel provides a relevant example of tissue with a complex hierarchical structure possessing a dense organized architecture across various scales and thus serves as a suitable object for the experiment.^19^

Human dental enamel comprises nanoscale hydroxyapatite (HAp) crystallites coaligned into micron-scale rods and surrounding inter-rod regions.^4,7,15,16,20,21^ The structure of enamel ensures exceptional mechanical properties, e.g., high hardness and toughness,^22−24^ but it has a low resistance to acidic attack that causes demineralization of the enamel.^25−27^ The acidic environment may arise from the activity of bacteria (e.g., *Streptococcus mutans* and *Lactobacilli*) that can lead to dental caries^28−31^ that remain a global disease.^32,33^ Other mechanisms such as acidic drink consumption may lead to nonbacterial acid erosion.^34^ In both cases, acid exposure leads to preferential demineralization of the enamel and loss of material manifested across many different scales, down to the nanoscale.^7,16,35−38^ Several experiments have been carried out to investigate enamel demineralization either statically (carious or artificial demineralization)^15,16,25,26,39,40^ or dynamically during artificial demineralization.^25,37,39,41,42^ Various techniques such as electron microscopy, X-ray diffraction, radiography, and tomography were used and have shown that not only was there loss of material but also that the structural parameters such as crystallite size and texture were altered after demineralization and need to be assessed using different analytical techniques. In previous studies, there was no straightforward method for correlating data between modalities because of the use of setups that involved complex and slow changes in detectors and energies between the two modalities. This leads to potential misalignment of data and prevents the capture of useful information occurring at short time scales.^43^

The present paper describes a methodology for correlative analysis of X-ray imaging and X-ray diffraction data. The data analysis was carried out using collection pipelines developed at DIAD and a range of postexperiment data analysis software. A direct correlation was established between absorption in the tomography data and intensity changes in the diffraction data. In addition, the visualization of the diffraction gauge volume within the tomographic image was demonstrated, revealing additional correlative details. The coaligned nature of the multimodal data is of clear interest in several fields of research in material *operando* studies beyond those in dental research.

## Methods

### Sample Preparation

The samples were two anonymized human third molars, one affected by caries and the one with unaffected enamel, which were extracted for noncaries-related therapeutic reasons at the School of Dentistry, University of Birmingham (ethical approval obtained from the National Research Ethics Committee; NHS-REC reference 14/EM/1128/Consortium Reference BCHCDent332.1531.TB). Blocks, measuring one millimeter high, were cut from each tooth with a low-speed diamond saw as previously described.^26,41^ The presence of caries was confirmed by visual inspection by a dentist. Since the samples were anonymized, no information about the age, sex ethnicity, or dietary habits of the individuals was available. The carious sample is referred to as sample C1 and was used for static data analysis and development of the data analysis routines. The unaffected tooth is referred to as sample NC2 and was used for the *in situ* demineralization experiment.

For the *in situ* demineralization study, the sample block NC2 was covered with varnish to leave a window of exposed enamel (∼300 μm × 235 μm) and mounted inside a tube to allow immersion in the demineralizing solution. The sample preparation followed the methodology described previously.^41^ The window was made on the side of the tooth approximately a third of the distance from the top of the crown (occlusal bite surface) to the gum. The sample was initially placed in artificial saliva (0.7 mmol L^–1^ CaCl_2_, 0.2 mmol L^–1^ MgCl_2_, 4.0 mmol L^–1^ KH_2_PO_4_, 30 mmol L^–1^ KCl, and 20 mmol L^–1^ Hepes, pH 7.0^44^) and then in lactic acid solution (10% volume at pH 2.2^25^) for more than 13 h.

### Optical Microscopy

Sample NC2 was visualized to locate the window with optical profilometry (Alicona Infinite Focus profilometer, Bruker, Coventry, U.K.) before the DIAD experiment at DLS, leading to a view of the window shown in the Supporting Information (SI) Figure S1a, and b.

### DIAD Experiment

The DIAD beamline processed the image and diffraction data with its internal calibration and provided matched data in a unified coordinate system so that the registration process between coordinates provided by the beamline was the only additional step required. The setup is shown in SI Figure S1c.

Imaging data was acquired using scintillator-coupled optics and a PCO.edge 5.5 camera (Excelitas Technologies, Waltham, MA 02451), with a field of view of ∼1.38 × 1.16 mm, a pixel size of 0.54 μm, and a beam energy of 22 keV. The reconstruction of the 2D projections equivalent to radiographs (1801 projections, scan from 0 to 180°, exposure time of 1 s, step 0.1°, and dimension 2560 × 2160 pixels) led to 3D tomography data sets of 2560 × 2160 × 2560 voxels with a voxel size of 0.54 μm, i.e., significantly finer that the typical cross-sectional dimension of the enamel rods (∼5 μm). Twenty dark-field and flat-field images were acquired to correct the projections for the background.

The 2D WAXS patterns were acquired in transmission mode with the PILATUS3 X CdTe 2M detector (pixel size 172 μm × 172 μm, Dectris AG, Baden-Daetwill, Switzerland) at the energy of 18 keV. The WAXS data was calibrated with LaB_6_ leading to the determination of the sample-to-detector distance of 38.4 cm. The diffraction beam spot size was 25 μm × 25 μm.

Crystallographic details were obtained using diffraction mapping done for each sample on a grid of 20 × 20 points with 5 s collection time per point and 44 and 55 μm step size in the horizontal and vertical directions for sample C1 and 60 and 60 μm for sample NC2, respectively. Based on the diffraction beam spot size, there was no overlapping of adjacent points. The data collection was performed by rastering the beam and not the stage. This led to minor changes in the diffraction geometry that were corrected using the lookup table of beam positions and WAXS data from the LaB_6_ calibrant. Maps of the summed total intensity of the WAXS patterns were obtained, as well as the details of the enamel diffraction peak (002) with the study of the integrated intensity around the rings (texture on the diffractograms). Since imaging and diffraction data had a unified coordinate system, it was possible to link directly the specific diffraction raster position with the corresponding radiograph and voxel position. The extraction of the gauge volume analyzed with WAXS using the tomography was carried out by using the 2D WAXS map as a mask applied to the reconstructed 3D tomography image.

All analyses were carried out at room temperature. Sample C1 was analyzed in the tomography and diffraction mapping modes. For the dynamic *in situ* experiment, sample NC2 was analyzed with time-lapse alternation of diffraction and tomography acquisition. Tomography data were acquired over 20 data sets to follow the demineralization process. The cell filled with the acid solution was closed to use sample immersion into liquid without flow. The details regarding the tomography setup and analysis are available elsewhere.^41,45^

The diffraction and tomography analysis were carried out using a combination of DIAD in-house scripts and pipelines in combination with SAVU^46,47^ for tomographic reconstruction, Matlab (MathWorks, US), DAWN,^48,49^ ImageJ/Fiji,^50,51^ and Avizo (Thermo Fisher Scientific, US) were used for image analysis and the latter for 3D data set segmentation.^16,26^

Diffraction data analysis was performed using DAWN to integrate WAXS data and visualize the results. The diffraction patterns with Debye–Scherrer rings were azimuthally integrated to obtain 1D intensity plots as a function of the scattering vector *q*. Texture was analyzed from the intensity of the Debye–Sherer ring as a function of the azimuthal angle φ. Matlab and OriginPro (OriginLab Corporation, Northampton, MA) were used for 2D fitting analysis and plotting of the diffraction data as described previously.^15,39^ The crystal structure of HAp was rendered using VESTA software^52^ using the CIF file 203027 from the Inorganic Crystal Structure Database (ICSD).^53,54^ 3D rendering of the data from the correlative analysis from the two modalities was performed using Avizo.

**Figure 1:**
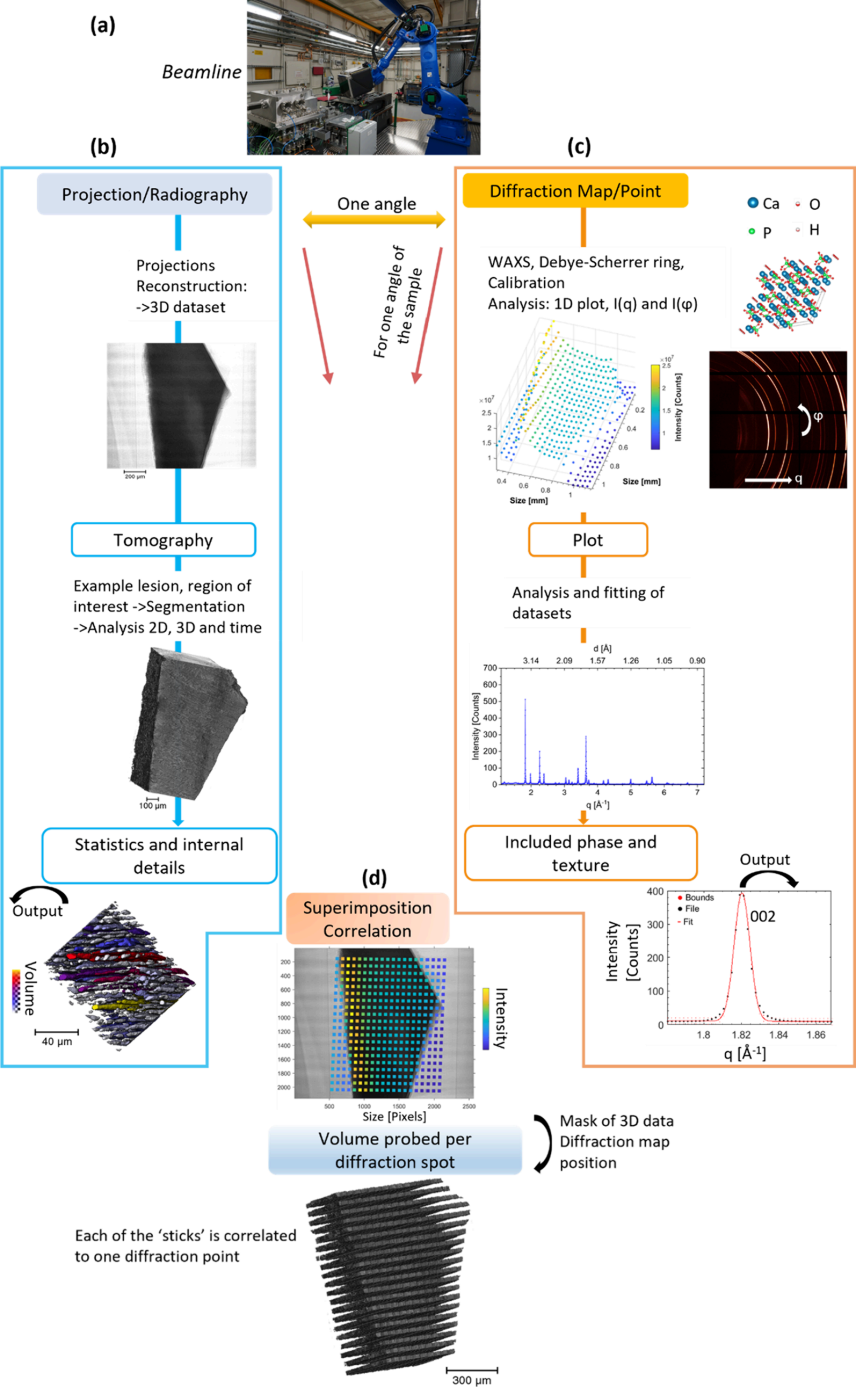
Schematic illustration of the workflow and outputs possible for the correlative analysis using the two DIAD modes. (a) Photograph of the beamline. (b, c) Workflow from the acquisition of the two modalities, WAXS and imaging, on the DIAD beamline. (b) Image analysis starting from one angle. Radiography imaging (equivalent to a projection) of sample C1 for the imaging mode. Tomographic reconstruction of the projection data sets allowed visualization and volume analysis of internal structure within the enamel slab of the tooth after computation. This is highlighted with 3D rendering of the tooth and statistical analysis on the volume. (c) From the same angle used for the projection described in (b), the corresponding WAXS map with origin of the signal the crystal lattices of the minerals in enamel (illustrated with the crystal lattice of HAp). The crystal structure of HAp was rendered with the software VESTA^52^ using the CIF file 203027 of the Inorganic Crystal Structure Database (ICSD).^53,54^ Each pixel from the WAXS map contributed to a WAXS pattern with Debye–Scherrer rings which were azimuthally integrated (azimuthal angle φ) to obtain 1D intensity plots as a function of the scattering vector *q* (or *d*-spacing or 2θ), shown for one pixel. Intensity of the (002) diffraction peak was obtained after fitting the peak. The (002) peak was used for texture analysis.^7,55^ (d) From the results of the two modalities, image of the superimposition of the two modalities from one angle and the volume rendering of the regions probed with the WAXS map. This described the outputs from both analyses and the correlative results with the localized analysis and the 3D rendering of the probed volume of the diffraction.

Figure 1 presents the overall objectives of the combined analysis carried out using imaging and diffraction.

### Scanning Electron Microscopy (SEM)

Sample NC2 after *in situ* demineralization during the DIAD experiment was visualized by SEM to reveal the sample surface topography. Secondary and backscattered electron images (SEi and BSi) were obtained using SEM Tescan Lyra 3 (Tescan, Czech Republic) with an accelerating voltage of 5 keV.

## Results and Discussion

**Figure 2:**
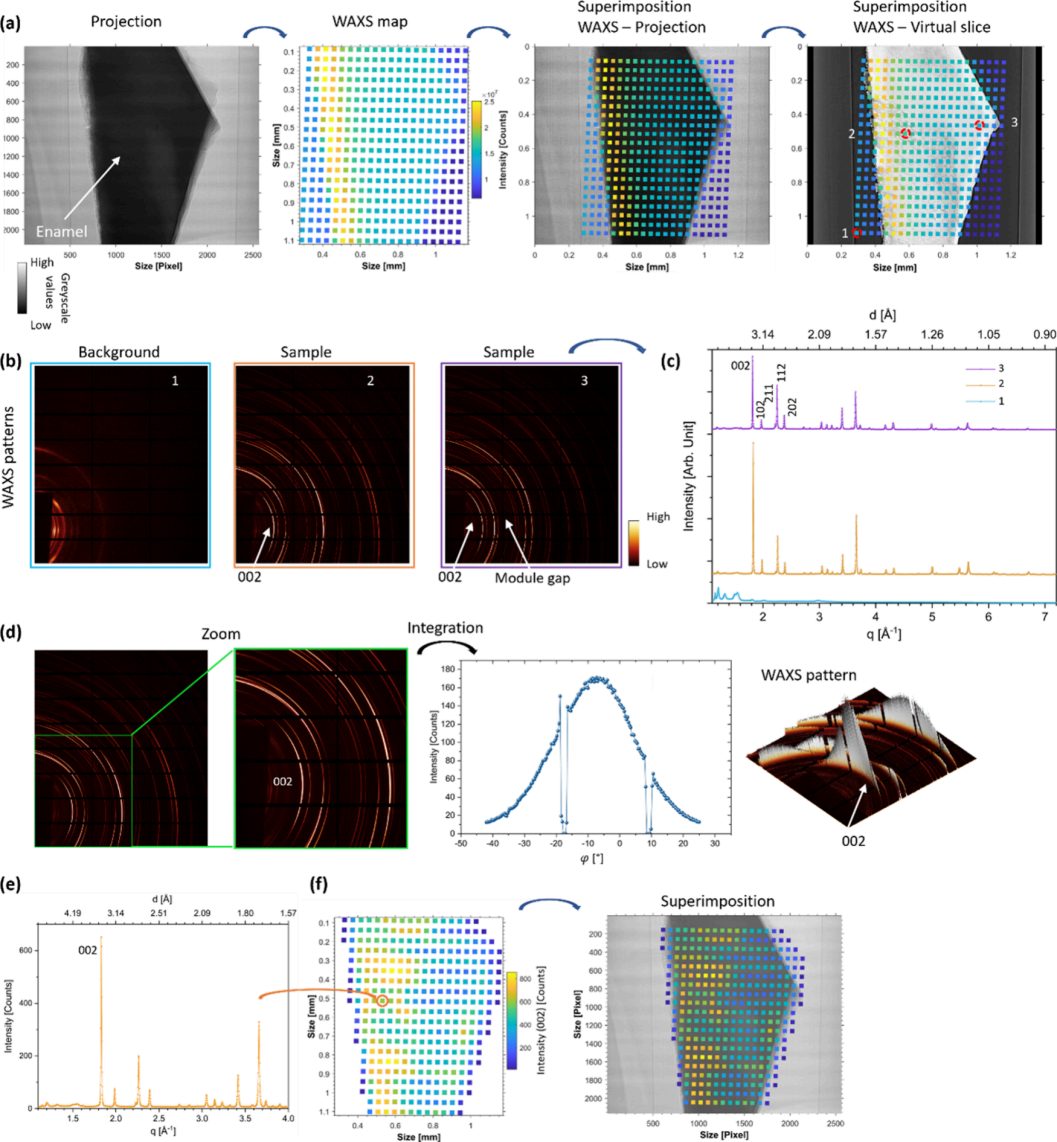
Analysis of human enamel caries, sample C1, correlating WAXS and attenuation in imaging acquisition. (a) A projection from sample C1 shows enamel on a bright background. WAXS mapping with the image of the sum of the intensity of each WAXS pattern and, finally, the two modalities registered with a projection and a virtual slice of the tomographic data. In the virtual slice, variation in the attenuation is suggested from the carious region. (b) WAXS patterns of the locations highlighted in (a) corresponding with carious enamel, noncarious enamel, and background from the gray scale. (c) A diffractogram of the WAXS patterns described in (b) clearly distinguishes between the background and the sample patterns. In the background location, plot number 1, the container where the sample was positioned likely contributed to the signal. (d) WAXS pattern with the plot after azimuthal integration along the (002) peak (azimuthal range of 67°), showing the intensity as a function of the azimuthal angle φ. In the WAXS pattern and plot, the drops of peak intensity seen are from the module gaps on the detector. (e) 1D plot showing a prominent (002) diffraction peak (additional details on the fitting are shown in SI Figure S2). (f) WAXS map of the intensity of the (002) peak and the superimposition on the sample projection.

**Figure 3:**
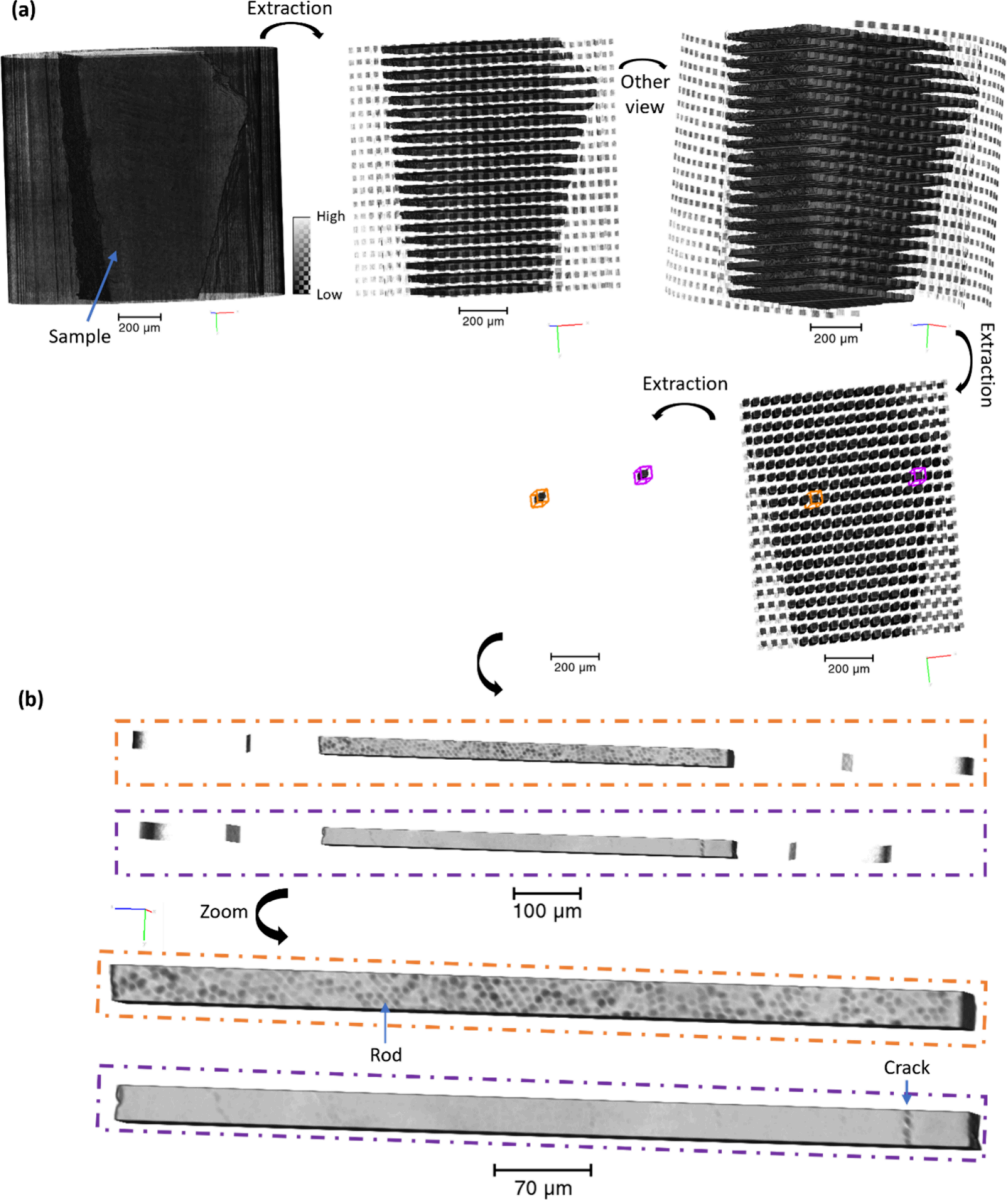
Tomography analysis of the enamel caries sample C1. (a) 3D rendering of the sample (2560 × 2160 × 2560 voxels, voxel size = 0.54 μm) and 3D rendering of the sample highlighting the probe volume acquired during the WAXS analysis, shown from two viewpoints. 3D rendering of C1 after segmentation of the WAXS acquisition locations with two highlighted locations described in Figure 2. (b) 3D rendering of the two locations showing variations in the structure, revealing the enamel rods in the carious region (orange) and a crack in the noncarious region (purple). The blocks were ∼630 μm in length, almost 10 times higher than the cross-sectional dimension which was 100 times higher than the pixel size of the tomography data set. The volume of the blocks illustrates the information about the structure acquired in the diffraction scan.

Tomography and diffraction mapping was successfully acquired from sample C1 allowing further correlative analysis from the unified coordinate system (Figures 1 and 2). Based on the coordinate registration from the two modes, the imaging projection and the WAXS map were correlated with the shape of the sample viewed from the contour of the sample suggested (Figure 2a). WAXS patterns from the diffraction map were extracted, and the texture was found from the computation of the azimuthal intensity profile from the integration of the diffraction arc located around the position of the (002) peak (Figure 2b and d)^7,55^ excluding the contribution from the module gaps of the detector. Following the integration of the 2D WAXS pattern, 1D diffractograms confirmed peaks from the HAp phase^54^ with the (002) peak at the sample locations compared to the off-sample sites (here referred to as background), as shown in Figure 2b and c. Differences in the peak intensities were found across the sample region analyzed, confirming the importance of localized inspection in the demineralized region of enamel (Figure 2e and f). The diffraction data set contains an average signal over the probe volume; individual scattering signals from voxels would only be obtained by performing a 3D diffraction-tomography experiment.^56^ The probe volume was obtained and is presented in Figure 3, using the registration of the coordinates, showing original data and visualization of acquired regions. This led to the visualization of the volumes analyzed within the diffraction map shown with volume renderings. One of the advantages of the registration of the two modalities (imaging and diffraction) is illustrated in Figure 3. The figure highlights the additional information gained from a 3D rendering of the volume extracted from the tomography data set, including the probed volume from the diffraction data acquisition locations. In the subvolumes analyzed in Figure 2, two subvolumes revealed differences in structure. The carious enamel was observed with the view of the rods from the variation of the gray values (Figure 3b and SI Figure S3), as previously observed from the demineralization of enamel,^16,26^ and the noncarious region with fewer details confirming the possibility of comparing the regions in enamel. A crack was also revealed, which was not easily seen with the projection data satisfying the interest and requirement of 3D analysis.

The proposed methodology can be transferred to analyze material samples to characterize grain phases in volumes with varying diffraction scattering, thus contributing to interpretation of the WAXS results. While this correlative approach was carried out in static mode, the same method was applied for a dynamic study of sample NC2 to evaluate the feasibility of following structure changes while submerged in liquid.

**Figure 4:**
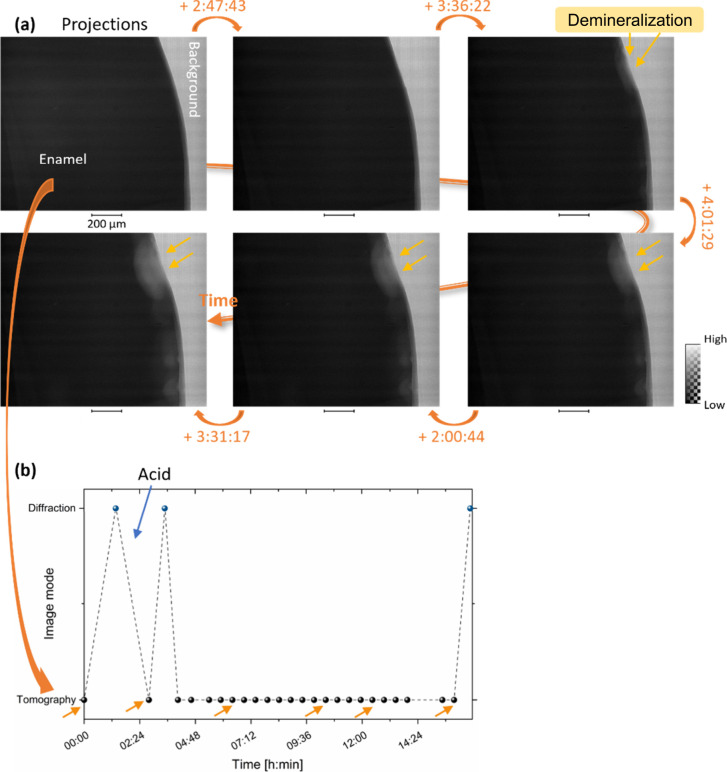
Time-lapse of the projections acquired during *in situ* demineralization of the normal enamel sample NC2. Images of projections were obtained at six different time points indicated by arrows in (b). Schematic of the data acquisition processes carried out during the experiment with time of acquisition from tomography and diffraction map. The arrows highlight the six projections shown. The time in (a) is annotated in the format hh:mm:ss.

**Figure 5:**
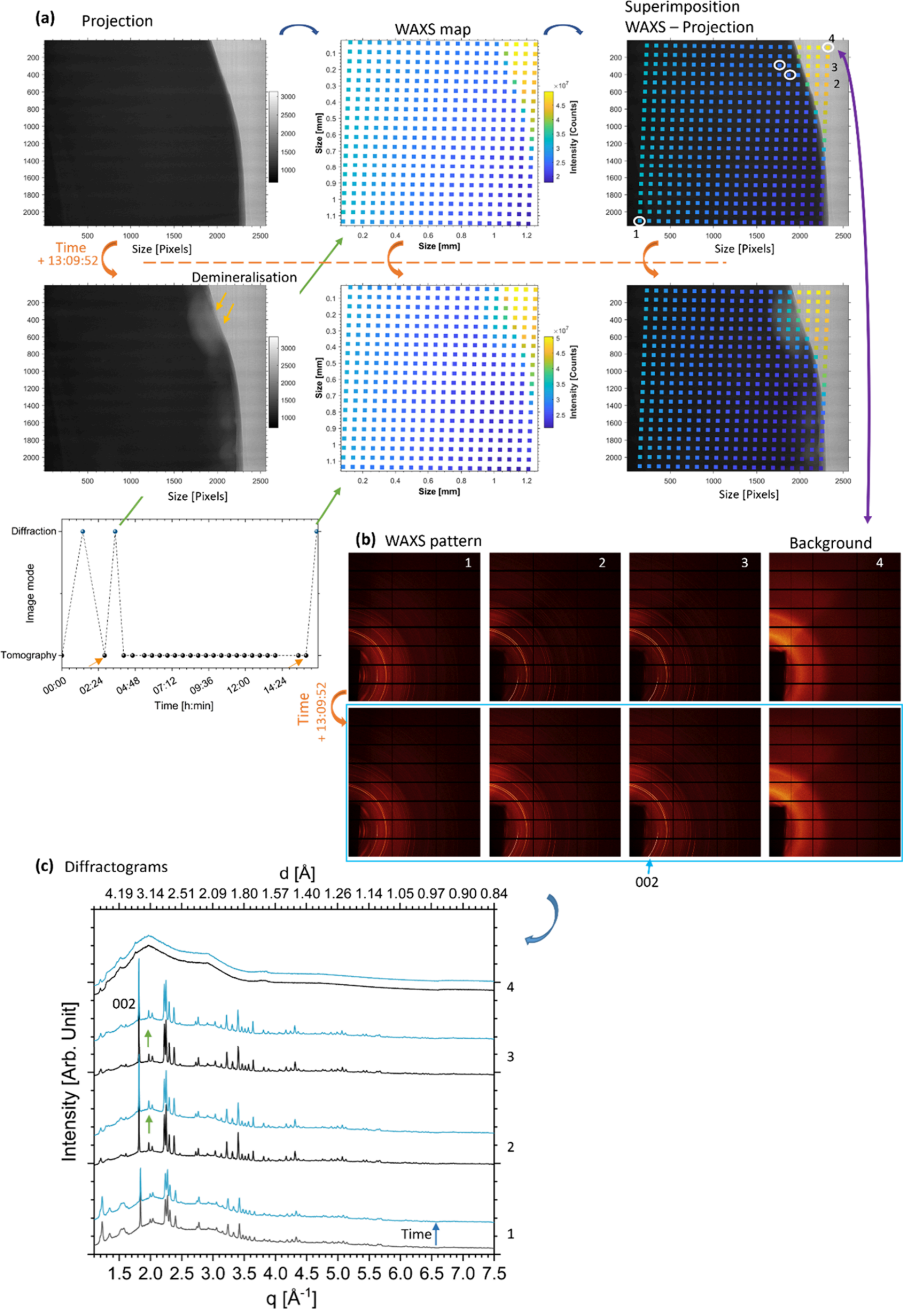
Correlative analysis of sample NC2 using imaging and diffraction. (a) Projections and diffraction acquisition point overlays of sample NC2 (orange arrows in the time trace indicate the time at acquisition of the projections) showing the background, enamel, and demineralization lesions (left); WAXS mapping and image of the sum of the intensity of each pattern at different times (center) and superimposition of the two modalities with the projection and the WAXS map (right). (b) WAXS patterns of the locations highlighted in (a). (c) The diffractogram of the WAXS patterns of the two maps for four locations described in (b) revealed a clear distinction between the patterns of the background and those from the sample. Similar as in Figure 2c, the container where the sample was placed likely contributed to the background. The plot of the diffractograms was offset for clarity in the visualization. Green arrows indicate the background in the diffractograms and the blue arrow the time (the plots in blue were from the time point after 13 h, 09 min described in (a)). The time in (a) is annotated in the format hh:mm:ss.

The noncarious enamel NC2 was analyzed during the *in situ* artificial demineralization process. Radiographs at different time points during exposure to the lactic acid solution showed the demineralization as changes in the gray scale, see Figure 4. There was a decrease in attenuation values in the demineralized regions. Imaging data sets did not provide information on the HAp crystal structure, but this could be assessed from the diffraction data. The WAXS mapping provided information on each scanning location and allowed tracking changes of the intensity in the patterns over time. Based on the map of the sum of the intensity of each WAXS pattern, intensity (sum of the detector pixel counts) changes were noticeable over time, particularly on the top right corner of the map of Figure 5a, which coincided with the demineralized region visualized with the imaging acquisition in Figure 4. Comparison of two time points with 13 h, 10 min of acid immersion between (the two time points were more than 2 and 14 h from the start of the experiment, see the time trace in Figure 5a, orange arrows) showed modified WAXS patterns in the demineralized region (also linked to the changes seen in the sum of the intensity of the diffraction data). The Debye–Scherrer rings in the sample showed evidence of HAp diffraction arcs in comparison to the background location (not on the sample) (Figure 5b). Sample texture was again observed based on the azimuthal intensity profiles obtained from the integration of the diffraction arcs at the Debye–Scherrer rings’ positions associated with the HAp phase. As outside of the specimen no HAp crystallites are present, the map produced by overlaying this value at the measurement positions facilitated visualization of the contour of the sample. The background in the intensity profile in the diffraction pattern was strong with a shoulder in the spectrum (Figure 5c) and was due to the presence of the liquid in the study of NC2 in comparison with the dry analysis of C1. At point 1 on Figure 5a far from the unvarnished window, the patterns from the two time points followed the same distribution of intensities, being less affected by the variation of liquid found in the demineralized region. HAp peaks were found with the prominent (002) peak assigned (Figure 5c). In the demineralized regions by comparing the same location at two time points, the diffractograms were observed to change. They revealed an increase in the background previously observed in the second time points after 13 h of immersion in acid, Figure 5c. Based on the image data, the origin of that increase was suggested to be the presence of liquid substituting the dissolved enamel (SI Figure S4). Demineralization of the enamel, as visualized in 3D, showed important alterations of its structure (SI Figure S4) with preferential demineralization of the rod over the inter-rod substance at the border of the lesion (SI Figure S4c). The 3D analysis, however lacked high-resolution topographic details, and hence SEM served as a complementary technique (SI Figure S5), which revealed a clear view of the sample and the presence of a cavity which was the result of the demineralization as well as cracks occasionally seen in SEM with a possible contribution of the dryness of the sample.

The approach described here applies to other fields in dental research previously studied without this correlative technique, including annealed samples,^57^ hard tissue remineralization,^40^ disease tissue,^58^ and mechanical properties, crack propagation, stress around cracks^59,60^ as well as other fields such as material sciences to study batteries, corrosion, and alloys.^7,61^ It is suggested that the possibility to probe volume and visualized internal details will help interpret WAXS data, including phases with different densities seen from the tomography and contributing to different peaks in diffraction data in comparison with the main matrix. This study gives new insights into how different techniques can provide complementary aspects of materials.

## Conclusions

Dental enamel was successfully characterized using a correlative analysis with imaging and diffraction in static and dynamic modes obtained at the DIAD beamline. The unified coordinate system for the two modalities allowed a direct comparison of imaging, radiography, tomography, and diffraction data. 2D WAXS and 2D–3D imaging modes showed good agreement in the shape of the sample with that of the WAXS maps. The probe volume acquired with WAXS was “revealed” with tomography, a step further in correlative techniques which reduced the amount of missing information and shows the importance of acquiring internal details as well as probing localized regions. The samples acquired revealed changes in the WAXS patterns and that the demineralization led to a variation of structure. The progression of the demineralization was visualized with time with preferential demineralization. Further analyses including larger samples would provide more statistical confidence to the data presented here. The two modalities can be set up to identify important supportive data from a sample, capturing information beyond traditional radiography. In addition to the dental application described, the techniques presented may be valuable in a wide range of research fields to study other materials’ structure statically and dynamically.

## Data Availability

Data collected and interpreted in this study is maintained by the authors and can be made available upon request.

## Supporting Information Available

Additional experimental details, including five figures of experimental results supporting the manuscript (PDF)
