# Supplementary material for: The DIAD Approach to Correlative Synchrotron X-ray Imaging and Diffraction Analysis of Human Enamel
Source: Chem Biomed Imaging. 2024 Mar 8;2(3):222–32. doi: 10.1021/cbmi.3c00122 (PMC10966737; doi:10.1021/cbmi.3c00122)
Supplement: Supplementary file 1 — im3c00122_si_001.pdf [file im3c00122_si_001.pdf]

The DIAD approach to correlative synchrotron X-ray imaging and diffraction analysis of human enamel

Authors

Cyril Besnard*[a], Ali Marie[a], Sisini Sasidharan[a,1], Hans Deyhle[b,2], Andrew M. James[b], Sharif I. Ahmed[b], Christina Reinhard[b,3], Robert A. Harper[c], Richard M. Shelton[c], Gabriel Landini[c], Alexander M. Korsunsky[d*]

[a] Department of Engineering Science, University of Oxford, Oxford, Oxfordshire, OX1 3PJ, U.K.

[b] Diamond Light Source Ltd., Didcot, Oxfordshire, OX11 0DE, U.K.

[c] School of Dentistry, University of Birmingham, 5 Mill Pool Way, Edgbaston, Birmingham, West Midlands, B5 7EG, U.K.

[d] Trinity College, University of Oxford, Broad St, Oxford, Oxfordshire, OX1 3BH, U.K.

Email addresses:

cyril.besnard@eng.ox.ac.uk, ali.marie@eng.ox.ac.uk, sisini.sasidharan@eng.ox.ac.uk, hans.deyhle@unibas.ch, andrew.james@swansea.ac.uk, sharif.ahmed@diamond.ac.uk, christina.reinhard@manchester.ac.uk, R.A.Harper@bham.ac.uk, R.M.Shelton@bham.ac.uk, G.Landini@bham.ac.uk, alexander.korsunsky@eng.ox.ac.uk

* ***Corresponding authors***: Cyril Besnard, cyril.besnard@eng.ox.ac.uk

Alexander M. Korsunsky, alexander.korsunsky@eng.ox.ac.uk

Present addresses:

[1] Department of Materials, Imperial College London, SW7 London, U.K.

[2] University of Basel, Department of Biomedical Engineering, Allschwil, Switzerland.

[3] The University of Manchester at Harwell, Diamond Light Source, Didcot, OX11 ODE, U.K.

Supporting Information (SI)

Table of Contents

Figures

Supporting Fig. S1.	p. 3
Supporting Fig. S2.	p. 4
Supporting Fig. S3.	p. 5
Supporting Fig. S4.	p. 6
Supporting Fig. S5.	p. 7

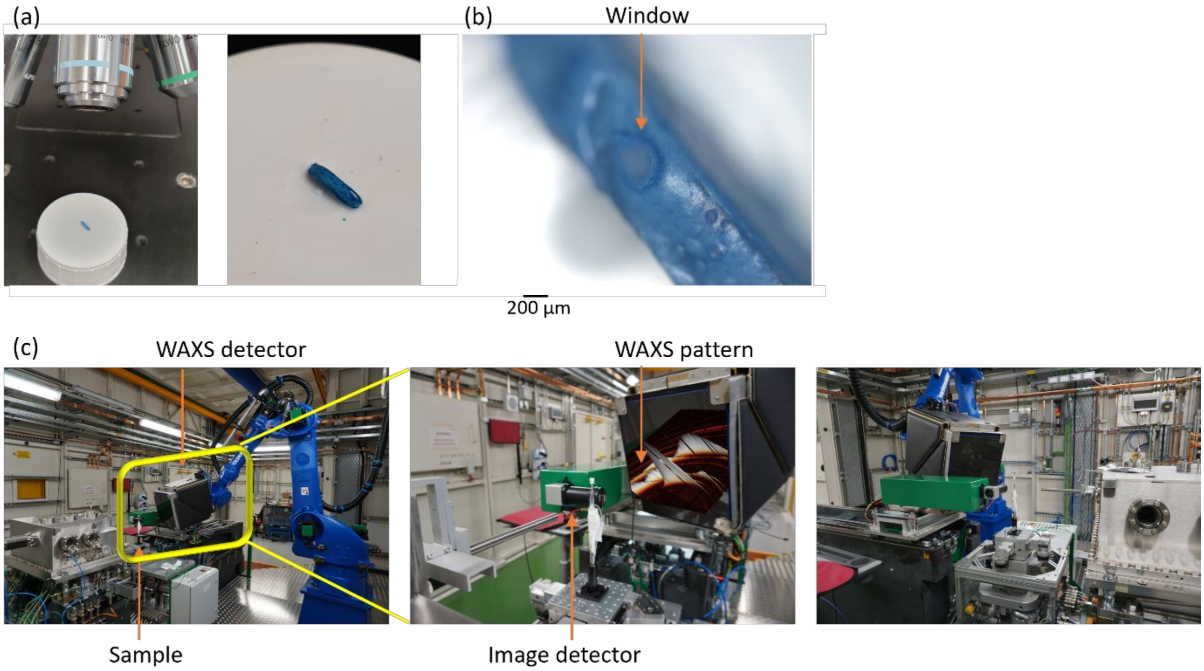

SI-Fig. S1. Description of the sample NC2 used for the dynamic study and the synchrotron setup. (a) Photographs of the sample prior to the experiment and (b) optical image of the sample with view of the window. (c) Photographs of the setup used for imaging and diffraction acquisition with the position of the detectors and the sample.

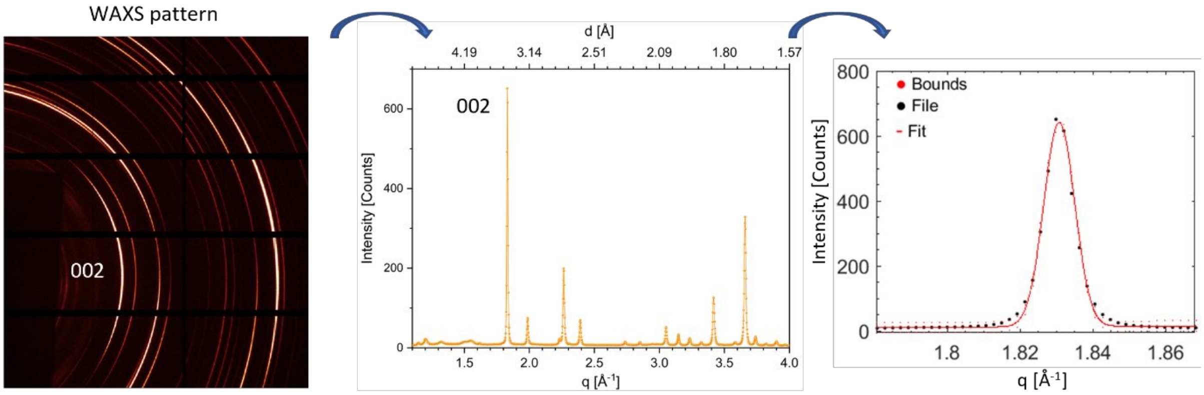

SI-Fig. S2. Additional WAXS details for the analysis of the sample C1. (a) WAXS pattern with the plot after azimuthal integration along the (002) peak (from an azimuthal range of 67°), showing the intensity as a function of the azimuthal angle φ. Analysis of the (002) diffraction peak with the fitting of the data based on the method in[1].

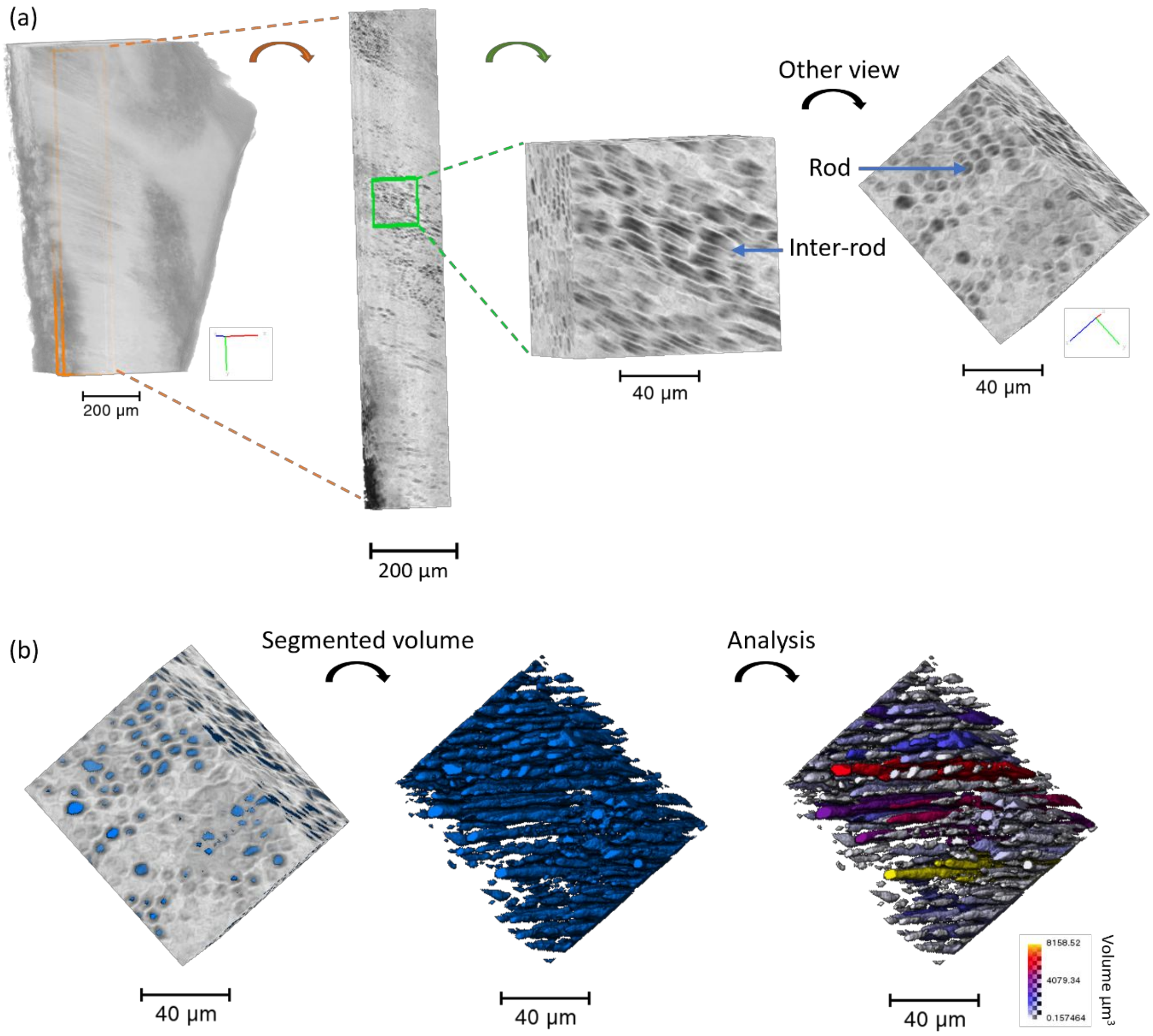

SI-Fig. S3. Details of the structure of enamel in the carious region of sample C1. (a) Volume rendering of the enamel and two regions of interest (343 × 2130 × 150 voxels and 200 × 200 × 150 voxels, voxel size of 0.54 µm). The view of the region of interest from two orientations showing the enamel rods and in (b) the segmentation of the rods superimposed with the volume of the enamel and the analysis of the volume of the segmented regions (additional details in the analysis of the volume in[2,3]). In the rods, decrease in attenuation values in the tomography data in comparison to the inter-rod regions from the demineralisation.

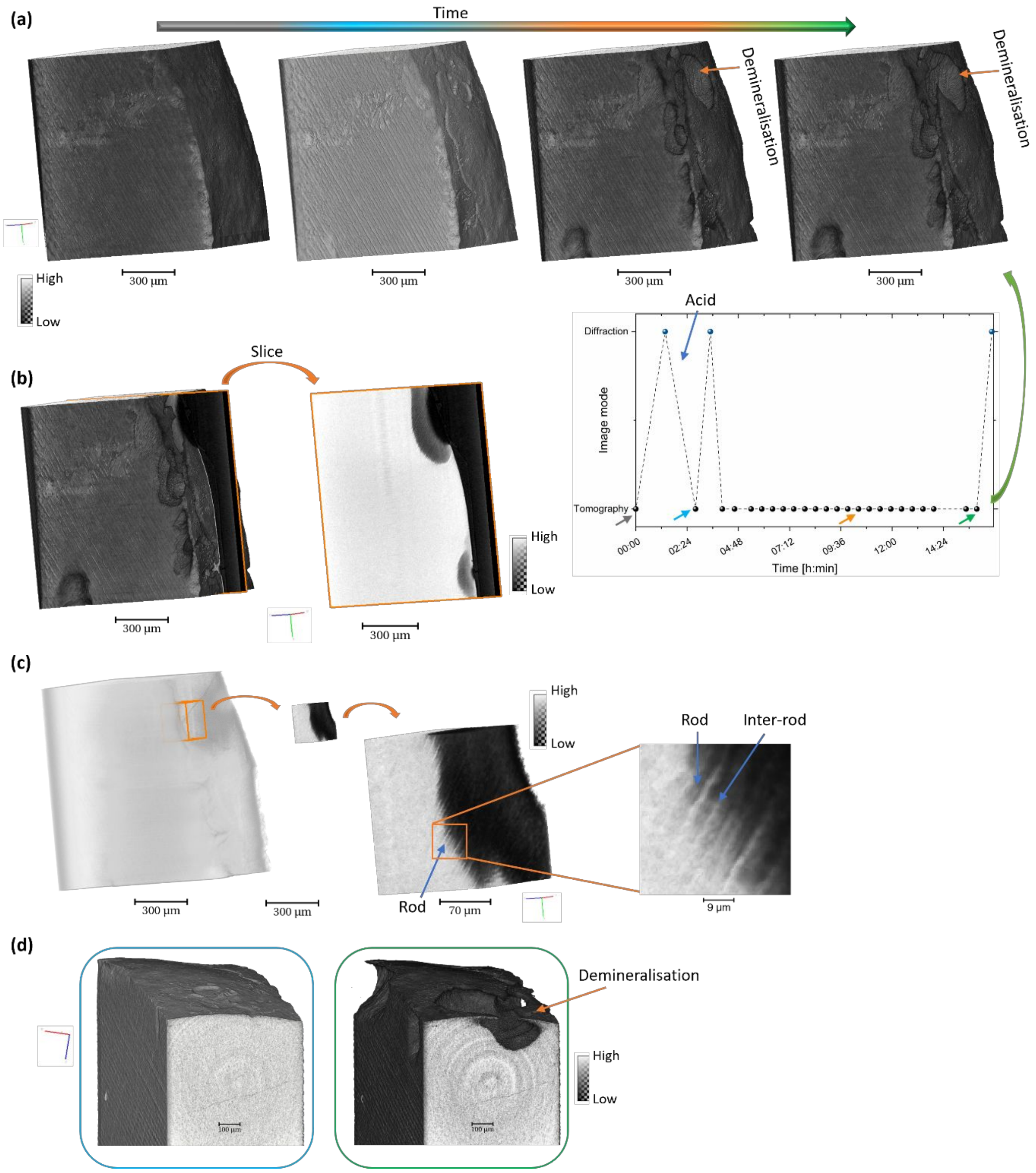

SI-Fig. S4. Tomography analysis of sample NC2. (a) Time-lapse of tomography analysis, time point described on the plot highlighted with arrows and colour code. (b) 3D rendering of the last time point and illustration of a virtual slice showing the variation of the grey value. A decrease in attenuation values in the tomography data was seen on the side of the sample. (c) 3D rendering of a region of interest showing the demineralised region and the view of rods and inter-rods highlighted in a small region. (d) 3D rendering of two time points from another view angle showing the progression of the lesion, colour code following the plot in (a).

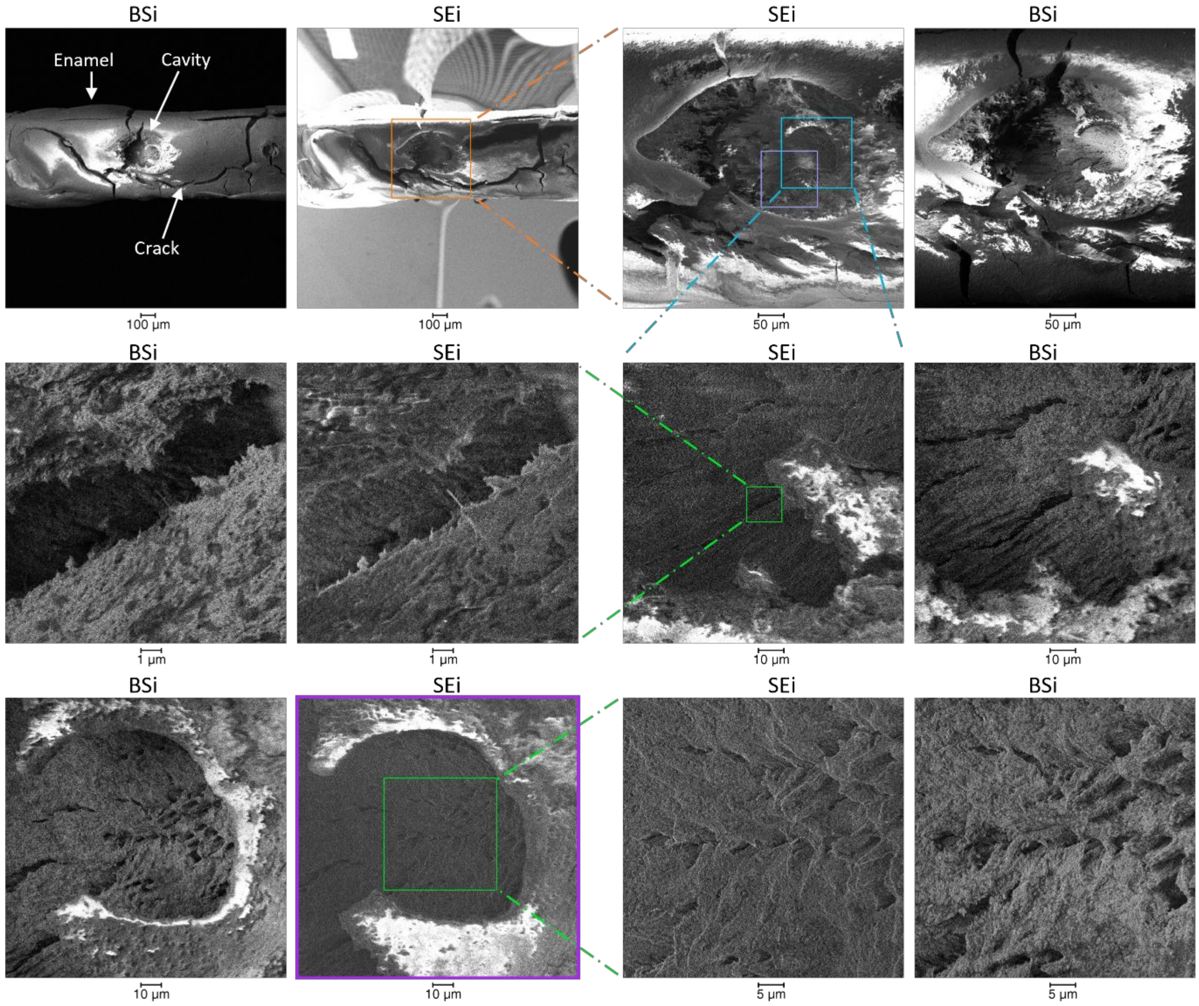

SI-Fig. S5. Scanning electron microscopy images of the sample NC2 after the X-ray synchrotron experiment. SEi and BSi acquired at various magnifications after the synchrotron experiment on the sample and immersion in liquid.

References

1 Besnard, C., Harper, R. A., Salvati, E., Moxham, T. E. J., Romano Brandt, L., Landini, G., Shelton, R. M. & Korsunsky, A. M. Analysis of *in vitro* demineralised human enamel using multi-scale correlative optical and scanning electron microscopy, and high-resolution synchrotron wide-angle X-ray scattering. *Materials & Design* **206**, 109739, doi:https://doi.org/10.1016/j.matdes.2021.109739 (2021).

2 Besnard, C., Marie, A., Buček, P., Sasidharan, S., Harper, R. A., Marathe, S., Wanelik, K., Landini, G., Shelton, R. M. & Korsunsky, A. M. Hierarchical 2D to 3D micro/nano-histology of human dental caries lesions using light, X-ray and electron microscopy. *Materials & Design* **220**, 110829, doi:https://doi.org/10.1016/j.matdes.2022.110829 (2022).

3 Besnard, C., Harper, R. A., Moxham, T. E. J., James, J. D., Storm, M., Salvati, E., Landini, G., Shelton, R. M. & Korsunsky, A. M. 3D analysis of enamel demineralisation in human dental caries using high-resolution, large field of view synchrotron X-ray micro-computed tomography. *Materials Today Communications* **27**, 102418, doi:https://doi.org/10.1016/j.mtcomm.2021.102418 (2021).
